# Development of a method for Making Optimal Decisions for Intervention Flexibility during Implementation (MODIFI): A modified Delphi study

**DOI:** 10.21203/rs.3.rs-3467152/v1

**Published:** 2023-11-02

**Authors:** Stephanie K Brewer, Catherine M Corbin, Ana A Baumann, Shannon Wiltsey Stirman, Janine M Jones, Michael D Pullmann, Aaron R Lyon

**Affiliations:** University of Washington School of Medicine; University of Florida College of Education; Washington University School of Medicine in Saint Louis: Washington University in St Louis School of Medicine; Stanford University School of Medicine; University of Washington College of Education; University of Washington School of Medicine; University of Washington School of Medicine

## Abstract

**Background.:**

Intervention adaptation is often necessary to improve the fit between evidence-based practices/programs and implementation contexts. Existing frameworks describe intervention adaptation processes but do not provide detailed steps for prospectively designing adaptations, are designed for researchers, and require substantial time and resources to complete. A pragmatic approach to guide implementers through developing and assessing adaptations in local contexts is needed. The goal of this project was to develop Making Optimal Decisions for Intervention Flexibility during Implementation (MODIFI), a method for intervention adaptation that leverages human centered design methods and is tailored to the needs of intervention implementers working in applied settings with limited time and resources.

**Method.:**

MODIFI was iteratively developed via a mixed-methods modified Delphi process. Feedback was collected from 43 implementation research and practice experts. Two rounds of data collection gathered quantitative ratings of acceptability (Round 1) and feasibility (Round 2), as well as qualitative feedback regarding MODIFI revisions analyzed using conventional content analysis.

**Results.:**

In Round 1, most participants rated all proposed components as essential but identified important avenues for revision which were incorporated into MODIFI prior to Round 2. Round 2 emphasized feasibility, where ratings were generally high and fewer substantive revisions were recommended. Round 2 changes largely surrounded operationalization of terms/processes and sequencing of content. Results include a detailed presentation of the final version of the three-step MODIFI method (Step 1: Learn about the users, local context, and intervention; Step 2: Adapt the intervention; Step 3: Evaluate the adaptation) along with a case example of its application.

**Discussion.:**

MODIFI is a pragmatic method that was developed to extend the contributions of other research-based adaptation theories, models, and frameworks while integrating methods that are tailored to the needs of intervention implementers. Guiding teams to tailor evidence-based interventions to their local context may extend for whom, where, and under what conditions an intervention can be effective.

## Introduction

Decades of research have established a wide variety of evidence-based prevention and intervention practices (EBP) for use across a range of healthcare domains. However, studies have also documented a persistent implementation gap in which EBPs are infrequently delivered at scale or with sufficient intensity to have their intended effects (Balas & Boren, 2000; Rolls Reutz et al., 2020; Williams & Beidas, 2019). Scholars have spent the past 15–20 years identifying implementation determinants (i.e., barriers and facilitators) and strategies (Cook et al., 2019; Krause et al., 2014; Powell et al., 2015), particularly at the intraorganizational and interorganizational levels (Dopp et al., 2019a; Lewis, Mettert, & Lyon, 2021). Relatively less attention has been devoted to optimizing implementation at the *intervention level.* The paucity of intervention-level research limits our understanding of intervention-level factors (e.g., intervention design quality) that might reveal novel paths to achieving quality implementation.

### Intervention Adaptation

There is often a mismatch between EBPs and the providers, clients, and service settings they aim to support (Damschroder et al., 2009). In response to this problem of contextual appropriateness or “fit,” EBPs are frequently modified to improve their functioning within a given implementation context (Lyon & Bruns, 2019). According to G. Moore et al. (2021), adaptations are intentional modifications made to EBPs to improve the intervention-implementation context fit; whereas modifications can be planned (e.g., changes made prior to intervention implementation) or reactive (e.g., intentional changes made in response to emergent need based on contextual fit of the EBP as delivered; Kirk et al., 2020; Miller et al., 2020). Though EBPs are routinely adapted to maximize fit with real-world settings, providers, and patients (e.g., Hall et al., 2016), such adaptations can be reactive in ways aligned more with implementer’s personal preferences for EBP use (Stirman et al., 2015) than with the intervention’s goal (Cooper et al., 2016), necessitating systematic ways to guide and understand the effectiveness of adaptations made across different phases of implementation.

As awareness of widespread adaptation has increased (Eisman et al., 2020; Lyon et al., 2019), the need to develop a full science of adaptation has become clear. One approach is to populate an “adaptome” to compile information about adaptation types and their respective impacts on implementation and intervention outcomes (Chambers & Norton, 2016). Implementation science has developed a number of taxonomies, models, and frameworks to systematize and guide efforts to identify, log, and assess the impact of adaptations. Both the Framework for Reporting Adaptations and Modifications-Expanded (FRAME; Stirman et al., 2019) and the patient-centered medical home (PCMH) adaptations model (Hall et al., 2017) characterize the who, what, when, where, and how of adaptations. While similar in their approach, FRAME offers more guidance on culturally responsive modifications whereas the PCMH adaptations model integrates implementer perceptions of the impact of modifications on implementation outcomes. The Model for Adaptation Design and Impact (MADI; Kirk et al., 2020) extends previous taxonomies (e.g., Stirman et al., 2019; Proctor et al., 2011) to propose associations between adaptation characteristics, mediating and/or moderating factors, and implementation and intervention outcomes. Additional models are emerging to assist researchers and practitioners in making decisions regarding whether or not to adapt and how to evaluate the adaptation process as it evolves (Miller et al., 2020).

These theories, models, and frameworks provide tools to accumulate knowledge about when intervention modification is indicated, what types of adaptations are made to interventions, and how to evaluate possible impacts on outcomes. An important next step is to develop methods of adaptation that build upon this foundation. Some existing models provide a direction for how to approach the intervention adaptation process. For example, the Dynamic Adaptation Process (DAP; Aarons et al., 2012) supports thoughtful intervention adaptation during four phases of implementation. Similarly, RE-AIM (Reach, Effectiveness, Adoption, Implementation, and Maintenance) is a comprehensive implementation framework that can be used to guide adaptations (Glasgow et al., 2020). The CENTER-IT (CENTERing multilevel partner voices in Implementation Theory) approach encourages researchers to incorporate stakeholder perspectives and consider the domains of the Consolidated Framework for Implementation Research (CFIR; Damschroder et al., 2009) when exploring possible adaptations (Trivedi et al., 2022). Similar to the DAP and RE-AIM, the ADAPT guidance (G. Moore et al., 2021) supports the entire implementation process and advances the science of adaptation by acknowledging adaptation as a crucial step and recommending important factors to consider when making adaptations (e.g., potential unintended consequences, costs and resources needed).

All of these frameworks can support intervention adaptation; but they require time, resources, and adoption of a large-scale implementation planning process that are not feasible in many settings. Further, beyond recommending convening a team to oversee this process and monitoring the need for *ad hoc* adaptations during implementation, none provide clear, detailed steps for how to adapt. What remains unarticulated are methods that “zoom in” on intervention modification and outline specific procedures for designing adaptations. These methods are especially needed in applied settings that lack the time, personnel, and other resources to launch a large-scale research-funded implementation planning process.

### Human Centered Design

The field of human centered design (HCD) offers methods that can augment existing adaptation processes. HCD is a field dedicated to bringing innovations into alignment with the users and settings where they will be deployed (Lyon, Brewer, & Arean, 2020; Norman & Draper, 1986). HCD methods can be used in efficient ways to elevate user perspectives, needs, and strengths. Fundamental to HCD is the expectation that engaging stakeholders in development or redesign processes should result in products that are more accessible, parsimonious, and usable. HCD has commonalities with other methodological approaches relevant to implementation such as community-based participatory research (CBPR; Oh, 2018), but in HCD user involvement is typically more targeted and time-limited (Lyon, Dopp et al., 2020). HCD complements implementation science’s multilevel frameworks by contributing well-specified approaches for engaging stakeholders, understanding user experience, and redesigning products (Chen et al., 2020; Lyon & Bruns, 2019b). Although HCD has been applied most commonly to the development of digital technologies, recent applications have made use of HCD principles and methods to improve the contextual fit of both psychosocial interventions and implementation strategies in adult and youth health services (Haines et al., 2021; Lyon, Coifman et al., 2021; Lyon, Koerner, & Chung, 2020; Lyon, Munson et al., 2019).

### Present Study

The goal of this research was to leverage HCD and develop a method for intervention adaptation that is tailored to the needs of intervention implementers (e.g., clinicians) and intervention decision makers (e.g., supervisors, program/site leaders) working in applied settings. To accomplish this goal, we focused on creating a method that (1) presents a focused set of techniques rather than a comprehensive collection of all possible ones; (2) optimizes feasibility and time/resource efficiency while remaining as scientifically rigorous as possible; (3) favors locally relevant and actionable information over widely generalizable knowledge; and (4) includes techniques from industry and HCD that can be executed rapidly and which center the experience of the end user. To ensure that the design process would not inadvertently modify an intervention in ways that eliminate its effectiveness, we included steps to identify the original intervention’s core functions (purposes) and forms (activities), as conceptualized by Jolles et al. (2019) and demonstrated by Kirk et al. (2021).

## Methods

To develop our method, we identified specific HCD techniques that could achieve the objectives of intervention adaptation steps drawn from a large-scale scoping study of adaptation frameworks. Escoffery et al. (2019) identified eight steps that were common across frameworks, and we selected the three steps most focused on designing adaptations: 1) decide what needs to be adapted, 2) adapt the original program, 3) test the adapted materials. Method development was completed via a series of literature reviews, team discussion meetings, and iterative revision. When the first draft of the intervention adaptation method was complete, we titled it *Making Optimal Decisions for Intervention Flexibility during Implementation* (MODIFI). To obtain feedback from people working in intervention adaptation research and practice, MODIFI was presented to a panel of experts in a two-round modified Delphi process.

### Participants

We employed a purposive snowball sampling procedure beginning with an initial list of experts generated by members of the study team. The initial list included people with expertise in intervention design, adaptation, and implementation. Efforts were made to recruit a sample of people with both research and applied professional roles. Potential participants were encouraged to identify peers with expertise in intervention adaptation and/or implementation science. Participants were offered group authorship on the paper publishing the final MODIFI method. We recruited a panel of 43 experts who provided feedback on MODIFI, each of whom was invited to provide feedback again in Round 2 (32 or 74.4% participated in Round 2). Participants were 65.12% female, average age was 44.98, and 81.40% were White (86.05% not Latino/a). Most participants held a Doctoral degree (95.35%), with an average 15.44 years implementing evidence-based programs or practices (range 5–35), and an average 8.77 years in their current professional role (range 1–35). Current professional roles were: 48.84% professor, 20.93% researcher; 11.63% clinician; 9.30% program/center director; 9.30% other.

### Procedures

**Figure 1:**
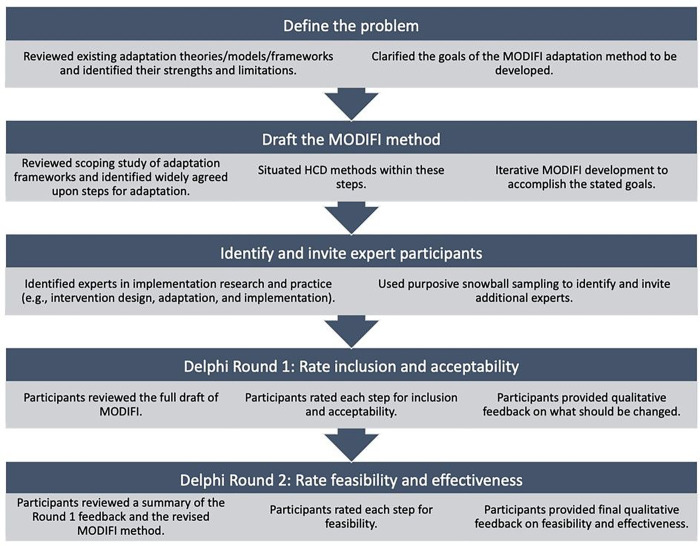
Delphi steps used in this study.

The Institutional Review Board at the first author’s institution approved all study procedures. This study employed a mixed-method design for the purpose of expansion, as qualitative methods were used to explain the results of quantitative methods (Palinkas, 2014). Quantitative and qualitative data collection occurred simultaneously (quan + QUAL), and analysis occurred sequentially (quan→QUAL). The Delphi technique was used to build consensus among a panel of experts, achieving convergence of opinion through multiple rounds of feedback (Hsu & Sandford, 2007). Figure 1 illustrates the steps of the modified Delphi process employed in this project.

The Round 1 survey first outlined the three phases of MODIFI (1. Decide what needs adaptation, 2. Adapt the EBR 3. Pilot test the adapted EBP) and allowed participants to review MODIFI in its entirety, including the techniques situated within each phase. Then, feedback was solicited on whether the three phases were acceptable for a guide that spells out how to make adaptations to an EBP For each step within each phase, participants were asked, “Do you think this step should be included in the adaptation how-to guide?” (answer choices: Essential, Optional, Inadvisable) and “Do you think this step is acceptable as it is currently written?” (answer choices: Yes, No [*If not what would you change to make this step acceptable?*]). Participants could offer additional thoughts and/or feedback at the end of the survey.

Round 1 quantitative data were analyzed using descriptive statistics examining the proportion of responses across categories. Round 1 qualitative data were analyzed using conventional content analysis, with the aim of understanding the perspectives of panel members without imposing preconceived categories onto the feedback (Hsieh & Shannon, 2005). The first author reviewed all responses and identified key concepts within each response, then grouped together responses that conveyed similar concepts (i.e., themes). Findings were discussed with the last author, and MODIFI was revised based on discussions of themes derived from qualitative analysis and iterative MODIFI redesign. Following a series of meetings and MODIFI revisions, a new version of MODIFI was developed.

The Round 2 survey began by specifying the intended primary users for MODIFI (intervention implementers and intervention decision-makers working in applied settings), as determined based on Round 1 feedback, as well as the intended primary use (streamlined methods optimized for feasibility while still being as scientifically rigorous as possible). A summary of the Round 1 feedback that was used to revise MODIFI was presented. Participants were asked to narrow the focus of any remaining recommendations to those that were crucial to ensure MODIFI’s feasibility and effectiveness. The survey instructed participants to review the contents of the revised MODIFI method so they could provide feedback on each part with an awareness of the whole. Then, for each part of revised MODIFI, feedback was solicited on whether there were any further revisions that were crucial for MODIFI’s feasibility and effectiveness. The Round 2 quantitative ratings (feasibility) changed from the Round 1 ratings (inclusion and acceptability) to reflect the clarified priorities of the MODIFI method based on panelists’ feedback. For the detailed steps of MODIFI, participants were asked, “Regarding this step, what do you think is its feasibility of use in applied contexts?” (answer choices: Completely infeasible, Somewhat infeasible, Neither infeasible nor feasible, Somewhat feasible, Completely feasible). Participants were asked to offer additional thoughts and/or feedback at the end of the survey. Round 2 feedback was used to develop the final version of MODIFI for dissemination. Data analysis following Round 2 employed the same procedures as Round 1.

## Results

Below, we first summarize the results of each round of the modified Delphi process and how it supported MODIFI development. Then, we present the outcome of this development process–the final MODIFI method (see Additional File 1).

### Round 1

The Round 1 survey focused on inclusion and acceptability. Most participants (61.4%) answered that the three MODIFI phases were acceptable for an adaptation how-to guide, while 38.6% answered that these overarching phases were unacceptable and provided written responses describing what they would change. For a full summary of the original MODIFI components and revision decisions based on Round 1 results, see Additional File 2.

For the specific MODIFI steps within each phase, participants rated inclusion favorably, with steps rated as “Essential” by 83.96% of participants on average, depending on the step (SD = 0.16). One step was rated as “Essential” by less than 50% of participants, and it was removed. In addition to inclusion, participants rated the acceptability of each specific MODIFI step. Steps were rated as “Acceptable” by 51.28% of participants on average, depending on the step (SD = 0.12). Given that almost half of participants considered most steps to be unacceptable as currently written, all steps were revised.

Acceptability ratings were further interpreted based on participants’ qualitative feedback. A summary of the Round 1 qualitative feedback can be viewed in Additional File 3. MODIFI was heavily revised based on this feedback, particularly the steps with lower ratings of acceptability. Following Round 1, MODIFI was streamlined, offering a narrower range of techniques. The revised version of MODIFI was designed to emphasize the creation of internally valid, locally actionable knowledge (which tends to be a more common focus in industry than in traditional academic research) and improve MODIFI’s feasibility for use in applied settings by intervention implementers (e.g., clinicians) and/or intervention decision-makers (e.g., supervisors, program/site leaders).

### Round 2

**Figure 2:**
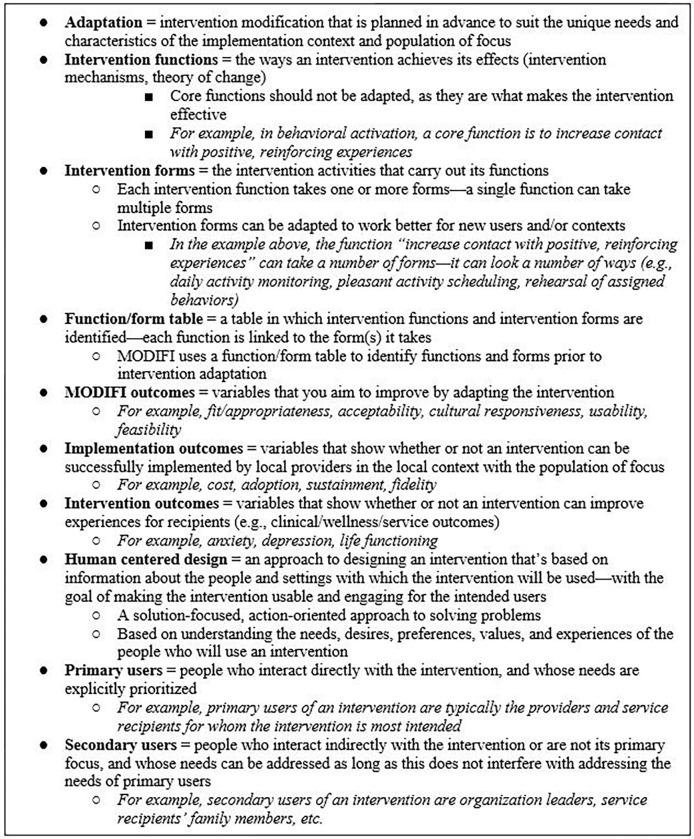
MODIFI definitions. *Note.* Definitions were based on the following sources: adaptation (Movsisyan et al., 2019); intervention functions, intervention forms, function/form table (Perez Jolles et al., 2019); implementation outcomes (Proctor et al., 2011); human centered design, primary users, secondary users (Dopp et al., 2019b).

For a summary of the revised MODIFI components and final revision decisions based on Round 2 results, see Additional File 4. The revised version of MODIFI participants rated in Round 2 was bookended by an introduction (MODIFI overview, definitions [see Fig. 2], prerequisites) and guidance for what users should do after completing the MODIFI process. Revised MODIFI comprised three steps: learn about the users, learn about the local context, and identify key information (Step 1); adapt the intervention (Step 2); and evaluate the adaptation (Step 3). After Step 3, MODIFI users were encouraged to return to earlier steps if the adaptation required revision to achieve the desired outcome.

Participants commented on a MODIFI phase or step if they believed it required additional revision. Higher rates of comments indicated where to apply additional revisions. Round 2 participants disproportionately commented on the introduction overview (59%) and definitions (66%), and two Step 1 components: learn about the users (53%) and identify key information about the intervention (78%). Additionally, participants rated each step’s feasibility of use in applied contexts, with all revised MODIFI components considered somewhat or completely feasible.

**Figure 3:**
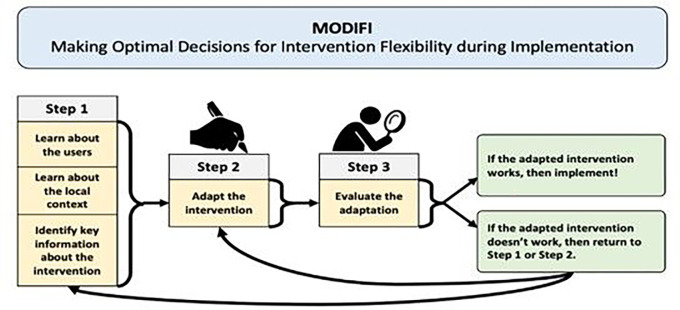
Final MODIFI steps.

Similar to Round 1, Round 2 qualitative feedback was used to inform the final MODIFI revisions. A summary of the Round 2 qualitative feedback can be viewed in Additional File 5. Following Round 2, there were fewer recommended revisions, and these revisions were relatively minor in scope. Round 2 changes largely surrounded operationalization of terms/processes and the sequencing of content. In response to the Round 2 feedback, the final MODIFI presents a simplified figure illustrating its overall process (Fig. 3); front loads content regarding participatory co-design methods; includes newly clarified definitions of several key concepts (including an example of an intervention function/form table); instructs participants in how to consider and respond to potential unintended consequences of adaptation; and emphasizes iterative evaluation and development both within MODIFI and following its final steps. Additional small-scale changes were made in response to minor feedback.

### The Final MODIFI Method

Round two feedback gave rise to the final version of MODIFI. The steps of MODIFI (see Fig. 3) are summarized below and presented in full in Additional File 1. MODIFI is used to make adaptations to *part of an intervention—not* to complete whole-intervention redesign. If the person/people applying MODIFI want to make multiple adaptations, it is recommended to complete the MODIFI steps for each adaptation (either one after another or at the same time in separate processes). MODIFI’s techniques can be carried out in many ways—to match the needs and resources of local settings, there are no prescribed numbers of participants, numbers of data collections, or time periods for data collection. MODIFI is a method that provides both structure and flexibility, because it is designed to be useful for a range of people and settings. For an illustrative case example describing what it looks like for people working in an applied setting to apply the MODIFI method for intervention adaptation, see Additional File 6.

**Figure 4:**
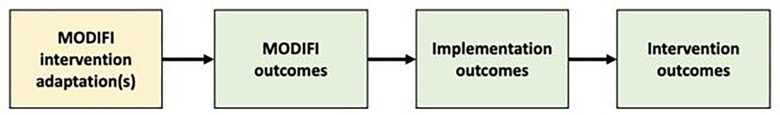
MODIFI logic model.

Unlike broader implementation frameworks, MODIFI is designed to “zoom in” on intervention modification and outline clear steps for how to design adaptations. Because MODIFI is a method to be used at a particular point in the implementation lifecycle, its application carries several preconditions. First, MODIFI users should already have selected an intervention to implement that addresses a problem for the population of focus, that relevant stakeholders believe is (or has the potential to be) appropriate, and ideally that has evidence for its effectiveness. Second, users have determined that adaptation is necessary (Baumann et al., 2015; Miller et al., 2020)—the original intervention cannot be implemented successfully due to potential problems with MODIFI outcomes (e.g., fit/appropriateness, usability, cultural responsiveness; see Figs. 3 and 4) and/or implementation outcomes (e.g., low fidelity, high cost). These outcomes are prioritized because they represent a causal chain. The goal of intervention adaptation is to improve the fit such that EBPs are more feasible, usable, culturally responsive, etc., and thus are more likely to be implemented with quality. Third, the user has formed a team to support intervention adaptation (G. Moore et al., 2021). Adaptation teams work best when they contain a mix of people in different roles (Aarons et al., 2012); specifically, primary users, people with expertise in the intervention (or the topic/problem it addresses), and people with expertise in intervention adaptation methods. When this is infeasible, MODIFI may be used cautiously by an individual intervention implementer or decision-maker.

### MODIFI Step 1: Learn About the Users

Step 1 of MODIFI has three components that can be completed in any order or simultaneously–learn about the users, learn about the local context, and identify key information about the intervention. Step 1 involves taking an applied anthropological approach (Hamilton & Finley, 2019)–listening, observing, and understanding experiences of the intended users within the local context–and additionally identifying the intervention’s functions and forms so the intervention can be adapted while maintaining effectiveness (Kirk et al., 2021). Learning about the users requires the person/people applying MODIFI to identify who will be the “users” of the adapted intervention (e.g., providers, service recipients; Lyon et al., 2019). At times, the person/people applying MODIFI are primary or secondary users themselves. Multiple users can be considered simultaneously by integrating information about their needs. After users have been identified, the person/people applying MODIFI conduct interviews asking users about their needs and assets related to the intervention and the topic/problem it addresses (Dopp et al., 2019b). Users might rank their unmet needs in order of priority to guide decision-making when designing adaptations. When needs conflict within or across user groups, needs should be prioritized in order of proximity to the intervention (e.g., primary users before secondary users). If needed, professional experience and research literature can be used to elaborate upon what is learned from users. After completing this component of Step 1, the person/people applying MODIFI should have a list of the highest priority unmet user needs to inform intervention adaptations in Step 2.

### MODIFI Step 1: Learn About the Local Context

Another component of Step 1 is to learn about the local context. First, the person/people applying MODIFI identify which aspects of the context (e.g., workflow, routines) are most likely to impact the intervention’s implementation. Then, in the context where the adapted intervention will be implemented, observations are conducted to gather information about the identified factors (Hamilton & Finley, 2019). What gets observed depends on the reasons that adaptation is needed to improve intervention-context fit, so the person/people applying MODIFI identify which aspects of the context they need to learn about in order to address the “WHY” of adaptation (J. E. Moore et al., 2021). Observations can be conducted efficiently by selecting aspects of the context that are practical to observe (e.g., physical location, working hours). The goal is to remain unobtrusive but not necessarily trying to be invisible–observers can be friendly, ask questions, reassure people that they are there to learn (not judge), and respect confidentiality (Dale et al., 2013). After completing this component of Step 1, the person/people applying MODIFI should have a list of aspects of the local context that may interfere with intervention implementation.

### MODIFI Step 1: Identify Key Information About the Intervention

Another component of Step 1 is to identify key information about the intervention. To adapt an intervention while retaining/maximizing its effectiveness, the person/people applying MODIFI must understand how it works. This can be accomplished via a function/form table that maps out how the intervention achieves its effects and is used to identify what can and cannot be changed during adaptation. Often core functions are not articulated by intervention developers but can be identified by creating a table with three columns: 1. Problems, 2. Functions, and 3. Forms (Kirk et al., 2021; Perez Jolles et al., 2019). Using the intervention materials (e.g., manual, website), consultation with intervention developers and/or experts, professional experience, and the research literature, the columns are populated with information including the problems the intervention aims to solve, the intervention’s functions–the goals or ways the intervention solves each problem–and the form(s) that each function takes within the intervention (e.g., intervention activities; see Table 1). With the function/form table completed, the person/people applying MODIFI will have a list of intervention functions (how the intervention solves problems)–these should remain unchanged in Step 2, and intervention forms–these may be adapted in Step 2. In the adapted intervention, each function is represented in at least one form.

### MODIFI Step 2

Step 2 of MODIFI uses a co-design method to adapt the intervention’s forms while leaving the functions intact. Co-design involves partnership between members of different groups to explore challenging problems and identify solutions (Metz et al., 2019). First, the person/people applying MODIFI identify who will participate in the co-design sessions. If possible, they should include at least one person with each of these roles: primary user, expert in the intervention (or the topic/problem it addresses), and expert in intervention adaptation methods. When this is not feasible, they should consider which viewpoints may be absent from the team and do their best to elevate those viewpoints as they present the information gathered during Step 1. When the group is assembled, they engage in co-design sessions (in-person or online), where they collaborate to: 1. understand the problem(s) to be solved through adaptation, 2. generate possible solutions, 3. co-create adaptations that solve the identified problem(s), 4. consider possible unintended consequences, and 5. iterate until the adaptation is ready for evaluation. In MODIFI each of these co-design aims is accompanied by a list of techniques. First, to understand the problem(s) to be solved, the person/people applying MODIFI present the information gathered in Step 1 (e.g., highest priority unmet user needs, aspects of the local context that may interfere with intervention implementation, and intervention functions), and the user/stakeholder members of the co-design sessions present information about their experiences. Then, to generate possible solutions, all co-design members contribute to brainstorming solutions to the problem(s) that they hope to solve through adaptation (IDEO, 2015). After brainstorming possible solutions, co-design members decide collaboratively which solutions (i.e., intervention adaptations) to select for co-creation. To create adaptations that solve the identified problem(s), co-design members draft intervention adaptation(s), during which they make sure that each intervention function is represented in at least one form (referring to the function/form table from Step 1). Then, co-design members iterate—co-creating further adaptation drafts, building upon each version by asking themselves, “How could this solution be just a little bit better?” (IDEO, 2015).

Next, co-design members reflect on potential impacts of the drafted adaptation(s) by discussing these questions (Kirk et al., 2020): Is this adaptation designed with specific goals in mind? Is this adaptation aligned with intervention core functions? And could there be unintended negative impacts of this adaptation (e.g., on adoption, cultural responsiveness, feasibility, cost)? Then, co-design members discuss possible negative impacts (e.g., increasing an EBP’s acceptability may reduce its effectiveness if the adaptation alters the EBP’s core functions; Kirk et al., 2020), the likelihood of negative impacts, and their severity, then consider whether these can be offset by positive impacts on other outcomes. Based on the findings of the impact analysis, further iteration may be warranted. In that case, the team co-creates further adaptation drafts. Finally, co-design members reach consensus by agreeing that the problem(s) have been solved well enough that the adaptation is ready for evaluation. After completing Step 2, the person/people applying MODIFI will know what adaptation is needed to match user needs/assets and local context realities.

### MODIFI Step 3

The goal of Step 3 is to generate evidence that is relevant to the identified users within the local context, not to collect evidence that is generalizable to other users and contexts (Daleiden & Chorpita, 2005). Thus, efficient, feasible, and locally appropriate evaluation methods are recommended. The person/people applying MODIFI should begin this step by thinking about what data they need to understand whether the intervention adaptation works for the identified users within the local context. If possible, they should include both quantitative and qualitative indicators of success (e.g., ratings of acceptability, quotes about cultural responsiveness, implementation outcomes). Ultimately, they should make decisions about what data they collect based on what’s feasible in their context, alongside what they learned from the users/context in Step 1 about the most important outcomes to maximize during intervention adaptation. Next, they should decide how they will measure the outcomes they have chosen, how often they will collect data, and what they need to see in order to conclude that the adaptation works for the identified users within the local context. These decisions are based on what they can actually track in their context. Data collection may be as narrowly scoped as a provider asking a service recipient if the adaptation is acceptable during each session while the adaptation is implemented or as complex as collecting data on multiple outcomes with multiple users over time before and after the adaptation is implemented.

### MODIFI Next Steps

Following the three steps of MODIFI, the person/people applying MODIFI should know whether the adapted intervention works for the identified users within the local context in a way that they find relevant and satisfying. If so, then they can implement the adapted intervention, and if resources allow, implement while collecting additional data (e.g., on the outcomes they have chosen, and/or on additional changes that occur during implementation). If the adapted intervention does not work for the identified users within the local context (or a subset of the identified users), the person/people applying MODIFI should either return to Step 1 if they need to learn more about the users, context, and/or intervention before further adaptation, or return to Step 2 if they know what further adaptation is needed, then continue from there.

## Discussion

We sought to develop MODIFI in response to a gap in the availability of pragmatic methods with which community-based teams can conduct implementation-explicit intervention redesign. MODIFI involves the systematic, prospective adaptation of intervention components to address intervention-level determinants. MODIFI leverages methods from HCD to gather locally-relevant, actionable information and design adaptations. We completed two Delphi rounds to gather feedback from experts in implementation science and HCD to refine the MODIFI method. In Round 1, most participants viewed all proposed components as essential but offered revisions to make them more acceptable. Round 2 emphasized feasibility, where ratings were generally high and fewer substantive revisions were recommended. Following Round 2, changes largely surrounded the overall framing of MODIFI, operationalization of its terms/processes, and sequencing of content.

As the implementation field grows, there are increasing opportunities for gaps to form between its research and practice components (Lyon, Comtois, Kerns et al., 2020; Westerlund, Nilsen, & Sundberg, 2019). While research has developed several models and frameworks to characterize stages of the intervention adaptation process (e.g., Hall et al., 2017; Stirman et al., 2019), pragmatic guidance for how implementers can design adaptations is lacking. In the absence of such scaffolding, implementers are likely to develop reactive, as opposed to proactive, adaptations that may not maintain the core functions necessary for the intervention to have its intended effects. MODIFI bridges implementation research and practice by offering a pragmatic and flexible method to empower community-based teams to lead this work. MODIFI can be paired with guidance on how to understand and evaluate intervention adaptations (see Kirk et al., 2020), thus allowing teams to engage in cycles of adaptation and evaluation to facilitate implementation of locally-tailored EBPs.

MODIFI extends the contributions of other adaptation theories, models, and frameworks while integrating methods that are tailored to the needs of intervention implementers. Existing adaptation frameworks are largely geared toward researchers and do not provide specific, feasible techniques. As MODIFI offers a rapid, resource-efficient method for designing adaptations, it represents an opportunity for implementation researchers to compare its effects with more resource-intensive models. A unique strength of MODIFI is its inclusion of HCD approaches for engaging stakeholders, understanding perspectives, and redesigning products in ways that elevate the voices of users. Increasingly, intervention and implementation researchers are drawing upon HCD concepts and techniques (e.g., Haines et al., 2021; Lyon et al., 2020a; Lyon et al., 2020b; Munson et al., 2022), and MODIFI provides an HCD-informed method with which to structure the application of these techniques to real-world intervention implementation problems. Thus, MODIFI may offer researchers new ways to approach intervention adaptations by prioritizing time- and resource-efficiency and user perspectives.

### Limitations

The limitations of this study include the lack of diversity on the panel of experts across multiple dimensions (65.12% female, 81.40% White, 95.35% Doctoral degree holders, 69.77% professor/researcher). Given the disproportionate number of academics on the expert panel, we made efforts to elevate feedback regarding the perspectives and needs of clinicians and other professionals working in applied settings. Another limitation may be the difference between ratings solicited in Round 1 (acceptability) versus Round 2 (feasibility) of the modified Delphi process. This reflected the shifting priorities of the MODIFI method but somewhat limited our ability to interpret differences in ratings across rounds. Finally, in response to feedback from stakeholders, MODIFI was developed to make adaptations to *part of* an intervention—not whole-intervention redesign—and to do so in efficient, cost-friendly ways. Because of this streamlining, it is not as comprehensive as other adaptation frameworks that involve a large-scale research-funded implementation planning process.

## Conclusions

Intervention-level determinants, including aspects of interventions that are not contextually acceptable, appropriate, or feasible, require more explicit attention in research and practice. To best meet the needs of local contexts, methods for identifying and addressing intervention-level barriers should be pragmatic and accessible to community practitioners. MODIFI empowers community-based teams with knowledge accumulated through rigorous implementation research by offering a pragmatic method that teams can use to proactively design adaptations to a prioritized EBP Guiding teams to tailor EBPs to their local context could extend for whom, where, and under what conditions EBPs can be effective. While MODIFI will benefit from future applications across multiple service settings and EBPs, our hope is that MODIFI becomes a tool for community-based teams to offer the version of EBPs that best meet the needs of the populations they serve.

## Figures and Tables

**Table 1:** Example Function/Form Table for TF-CBT.

Problems	Functions	Forms
•Posttraumatic symptoms •Dysfunctional trauma-related cognitions (i.e., negative beliefs about self, others, the world, & the future) •Reexperiencing the trauma (e.g., intrusive memories, dreams) •Heightened arousal & reactivity •Affective dysregulation •Avoidance of trauma reminders	•Change trauma-related cognitions •Improve emotion regulation •Decrease behavioral avoidance	•Cognitive coping skills, trauma narration & processing, psychoeducation •Affective modulation skills, relaxation skills, cognitive coping skills •*In vivo* mastery of trauma reminders, cognitive coping skills
•Depression symptoms •Feeling sad, empty, hopeless, worthless •Decreased interest in activities •Suicidal ideation	• Change trauma-related cognitions	• Cognitive coping skills, trauma narration & processing, psychoeducation

## Abbreviations

MODIFI: Making Optimal Decisions for Intervention Flexibility during Implementation
EBP: Evidence-based prevention and intervention practice
FRAME: Framework for Reporting Adaptations and Modifications-Expanded
PCMH: Patient-centered medical home
MADI: Model for Adaptation Design and Impact
DAP: Dynamic Adaptation Process
RE-AIM: Reach, Effectiveness, Adoption, Implementation, and Maintenance
CENTER-IT: CENTERing multilevel partner voices in Implementation Theory
CFIR: Consolidated Framework for Implementation Research
HCD: Human centered design
Quan: Quantitative
Qual: Qualitative
TF-CBT: Trauma-Focused Cognitive Behavioral Therapy
